# Supplementary material for: Coherent interaction-free detection of microwave pulses with a superconducting circuit
Source: Nat Commun. 2022 Dec 7;13:7528. doi: 10.1038/s41467-022-35049-z (PMC9729670; doi:10.1038/s41467-022-35049-z)
Supplement: Supplementary file 1 — Supplementary Information [file 41467_2022_35049_MOESM1_ESM.pdf]

Supplementary Information
Coherent interaction-free detection of microwave pulses with a superconducting circuit

Shruti Dogra, John J. McCord, and Gheorghe Sorin Paraoanu
QTF Centre of Excellence, Department of Applied Physics,
Aalto University, FI-00076 Aalto, Finland

(Dated: November 10, 2022)

This supplement more thoroughly explores the coherent interaction-free measurements presented in this work, and discusses how our coherent interaction-free detection scheme compares with the standard projective non-unitary case typically realized in quantum optical systems [1]. In particular, we compare efficiencies for both schemes up to $N = 25$, and further compare these two cases when dissipation is applied via the Lindblad master equation. We also present general analytical expressions for arbitrary N for our coherent protocol and various simulations in support of our claims. An alternative analysis of the coherent interaction-free detection protocol is developed by considering the quantization of the B-pulse, which bears the same results as the ones obtained from the semi-classical description. This exercise helps contribute to an in-depth understanding of the process. Finally, we provide a geometric representation of the detection process on the Majorana sphere. We begin by presenting detailed analysis of interaction-free measurements in general and its coherent counterpart.

SUPPLEMENTARY NOTE 1: FIGURES OF MERIT

We introduce the key figures of merit for the $N = 1$ case. For $N > 1$, they can be generalized in straightforward ways. The Positive Ratio PR $= p_0(\theta)/(p_0(\theta) + p_1(\theta))$ is a measure of the correct detection of a B-pulse with arbitrary strength (θ) and the Negative Ratio NR $= p_1(\theta)/(p_0(\theta)+p_1(\theta))$ is the incorrect non-detection of a B-pulse when it is applied with strength (θ). Special cases are defined as follows: FPR and TNR correspond to $\theta = 0$, while TPR and FNR correspond to $\theta = \pi$ for PR and NR respectively. In fact, PR(θ) effectively corresponds to the number of instances that report an interaction-free measurement of the B-pulse and NR(θ) are the inconclusive outcomes, where both of these quantities are obtained by excluding the situations where B-pulses are absorbed. In other words, for $N = 1$ and $\theta = \pi$ we have a 50% chance that the pulse is not absorbed. By postselecting over these cases, we find that we can either sucessfully detect the pulse (with 50% probability), or we cannot conclude anything (again with 50% probability). This is the meaning of TPR and FNR, see also Fig. 2 in the main text. The extension of this logic for the case of large N suggests that larger values of PR(θ) have direct correspondence with increasing probability of interaction-free/absorption-free detection of the B-pulse of strength θ.

	Predicted positive	Predicted negative
Actual positive	TPR $= \frac{p_0(\theta=\pi)}{p_0(\theta=\pi)+p_1(\theta=\pi)}$ (True Positive Ratio)	FNR $= \frac{p_1(\theta=\pi)}{p_0(\theta=\pi)+p_1(\theta=\pi)}$ (False Negative Ratio)
Actual negative	FPR $= \frac{p_0(\theta=0)}{p_0(\theta=0)+p_1(\theta=0)}$ (False Positive Ratio)	TNR $= \frac{p_1(\theta=0)}{p_0(\theta=0)+p_1(\theta=0)}$ (True Negative Ratio)

Supplementary Table I. The confusion matrix as defined for the coherent detection of π pulses. The concept is in fact applicable for the projective protocol as well, with the use of $p_{\rm det}$ and $1 - p_{\rm det} - p_{\rm abs}$ in place of p_0 and p_1 respectively.

Borrowing from the standard terminology of hypothesis testing, we can introduce the confusion matrix for our detection protocol. We indicate the presence ($\theta = \pi$) or absence ($\theta = 0$) of a B-pulse as positive or negative respectively. The elements of the confusion matrix are summarized in Supplementary Table. I. Specifically, a true positive (TP) is the correct detection of an applied π pulse (actual positive event), while a false positive (FP) is the incorrect prediction of the pulse when it has not been in fact applied (actual negative event). Strictly speaking, the complementary predictions are inconclusive in our case. However, for conformity, we will use the standard terminology of negative prediction to designate them, namely false negative (FN) and true negative (TN) for the cases when there was and was not a pulse present, respectively.

In analogy with the optical case [2, 3], we can also introduce the coherent interaction-free efficiency $\eta_c(\theta) = p_0(\theta)/[p_0/(\theta) + p_2(\theta)]$ as the fraction of pulses detected in an interaction-free manner while discarding the inconclusive results.

It is also important to emphasize the role of setting the Ramsey sequence such that, in the absence of the pulse, the final state is $|1\rangle$ and not say some superposition of $|0\rangle$ and $|1\rangle$. This ensures that, when finding the system in the state $|0\rangle$, we know with 100% certainty that the pulse was present; in other words, that FPR=0 and TNR=1 in the ideal case.

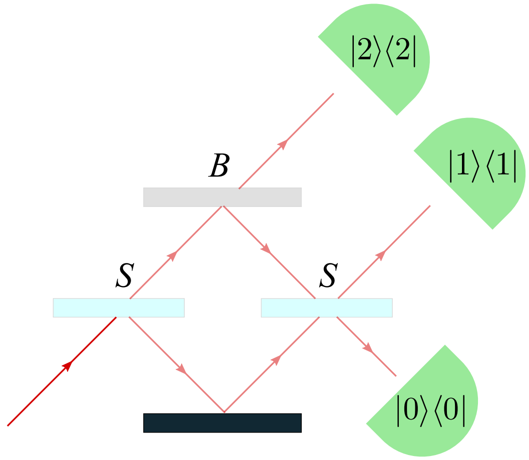

Supplementary Figure 1. Schematic of a generalized optical interaction-free interferometric setup, where the absorption probability can take values from 0% to 100%.

SUPPLEMENTARY NOTE 2: COHERENT VERSUS PROJECTIVE INTERACTION-FREE MEASUREMENTS

We discuss here the difference between the standard non-unitary (projective) interaction-free measurement and our approach. To make the connection clear, we start with the $N = 1$ case, for which simple analytical results can be provided.

A. $N = 1$ case

From the definitions in the main text we have

$$S_1 = \frac{1}{\sqrt{2}}\mathbb{I}_{01} - \frac{i}{\sqrt{2}}\sigma^y_{01} + |2\rangle\langle 2|, \tag{1}$$

$$B(\theta) = |0\rangle\langle 0| + \cos\frac{\theta}{2}\mathbb{I}_{12} - i\sin\frac{\theta}{2}\sigma^y_{12}, \tag{2}$$

where $\mathbb{I}_{kl} = |k\rangle\langle k| + |l\rangle\langle l|$ and $\sigma^y_{kl} = -i|k\rangle\langle l| + i|l\rangle\langle k|$.

The corresponding Mach-Zehnder interferometric setup for non-unitary interaction-free measurements is shown in Supplementary Fig. 1. The final experimental results are the events (clicks) recorded by the detectors $D_{0,1,2}$ modeled as projection operators onto the corresponding states, $D_0 = |0\rangle\langle 0|$, $D_1 = |1\rangle\langle 1|$, and $D_2 = |2\rangle\langle 2|$. By introducing a beam-splitter with finite reflectivity in the upper branch of the interferometer, we generalize the typical optical setups to the situation where the detector D_2 clicks only for a fraction of events.

To analyze what happens, let us first notice that in the absence of the B-pulse there is obviously no difference between the two approaches – in both cases the evolution operator is the unitary $S_1^2 = -i\sigma^y_{01} + |2\rangle\langle 2|$. But, in the presence of a B-pulse, the superposition created by S_1 gets modified to

$$B(\theta)S_1|0\rangle = \frac{1}{\sqrt{2}}|0\rangle + \frac{1}{\sqrt{2}}\cos\frac{\theta}{2}|1\rangle + \frac{1}{\sqrt{2}}\sin\frac{\theta}{2}|2\rangle. \tag{3}$$

The measurement operators associated with the detector $|2\rangle\langle 2|$ clicking (absorption) or not clicking (non-absorption) are the projectors

$$P_{\rm abs} = |2\rangle\langle 2|, \tag{4}$$

$$P_{\overline{\rm abs}} = |0\rangle\langle 0| + |1\rangle\langle 1|. \tag{5}$$

Thus, with probability $p_{\rm abs}(\theta) = \langle 0|S_1^\dagger B^\dagger(\theta)P_{\rm abs}B(\theta)S_1|0\rangle = (1/2)\sin^2(\theta/2)$ the state of the system collapses to $\left(1/\sqrt{p_{\rm abs}(\theta)}\right)P_{\rm abs}B(\theta)S_1|0\rangle = |2\rangle$ if a click is recorded by the detector $|2\rangle\langle 2|$; otherwise, with probability $p_{\overline{\rm abs}}(\theta) = \langle 0|S_1^\dagger B^\dagger(\theta)P_{\overline{\rm abs}}B(\theta)S_1|0\rangle = 1/2 + (1/2)\cos^2(\theta/2)$, the state collapses onto $\left(1/\sqrt{p_{\overline{\rm abs}}(\theta)}\right)P_{\overline{\rm abs}}B(\theta)S_1|0\rangle = \left(1/\sqrt{1+\cos(\theta/2)}\right)(|0\rangle + \cos(\theta/2)|1\rangle)$. Therefore, a non-absorption event has consequences: it confines the state to the $\{|0\rangle, |1\rangle\}$ manifold. For the case $\theta = \pi$, this confinement is onto the state $|0\rangle$ (we know for sure that the photon has traveled only in one branch of the interferometer), while the case $\theta = 0$ corresponds to a completely reflective beam-splitter B, which fully hides the detector $|2\rangle\langle 2|$, and as a result the equal-weight superposition of $|0\rangle$ and $|1\rangle$ is not affected.

Note here that we can define the POVM measurement operators associated with the ensemble beam-splitter B plus $|2\rangle\langle 2|$-detector from Supplementary Fig. 1 by $M_{\rm abs} = P_{\rm abs}B$ and $M_{\overline{\rm abs}} = P_{\overline{\rm abs}}B$, with the property $M^\dagger_{\rm abs}M_{\rm abs} + M^\dagger_{\overline{\rm abs}}M_{\overline{\rm abs}} = \mathbb{I}_3$, where $\mathbb{I}_3$ is the 3×3 identity matrix.

The density matrix after the second beam splitter can be found by again applying S_1 to the states written above. Therefore, the state at the output is

$$\left(\sin^2\frac{\theta}{4}|0\rangle + \cos^2\frac{\theta}{4}|1\rangle\right)\left(\sin^2\frac{\theta}{4}\langle 0| + \cos^2\frac{\theta}{4}\langle 1|\right) + \frac{1}{2}\sin^2\frac{\theta}{2}|2\rangle\langle 2|. \tag{6}$$

As a result, the probability of interaction-free detection is $p_{\rm det} = \sin^4(\theta/4)$ (detector $|0\rangle\langle 0|$ clicks) and the efficiency η, defined as the fraction of successful detections by excluding the inconclusive cases ($|1\rangle\langle 1|$ clicks) is

$$\eta = \frac{p_{\rm det}}{p_{\rm det} + p_{\rm abs}} = \frac{2\sin^4(\theta/4)}{2\sin^4(\theta/4) + \sin^2(\theta/2)}. \tag{7}$$

Consider now the coherent case. At the end of the protocol, the state is

$$S_1B(\theta)S_1|0\rangle = \sin^2\frac{\theta}{4}|0\rangle + \cos^2\frac{\theta}{4}|1\rangle + \frac{1}{\sqrt{2}}\sin\frac{\theta}{2}|2\rangle. \tag{8}$$

We can immediately verify that, by applying the same projectors $P_{\rm abs}$ and $P_{\overline{\rm abs}}$ corresponding to a measurement

of the state $|2\rangle$, we obtain precisely the result Eq. (6). We have $p_0 = \sin^4(\theta/4)$, $p_0 = \cos^4(\theta/4)$, $p_2 = (1/2)\sin^2(\theta/2)$ and the coherent-case efficiency is

$$\eta_c = \frac{p_0}{p_0 + p_2} = \frac{2\sin^4(\theta/4)}{2\sin^4(\theta/4) + \sin^2(\theta/2)}, \tag{9}$$

the same as Eq. (7). This is due to the fact that $S_1 P_{\rm abs} = P_{\rm abs} S_1$ and $S_1 P_{\overline{\rm abs}} = P_{\overline{\rm abs}} S_1$, so it does not matter when we record the result of the projection on $|2\rangle\langle 2|$.

In conclusion, for $N = 1$ there is no difference in the success/failure probabilities and the efficiency between the coherent and projective cases. The corresponding experimental results are shown in Supplementary Fig. 2, together with a comparison with the simulations and the ideal (decoherence-free) case.

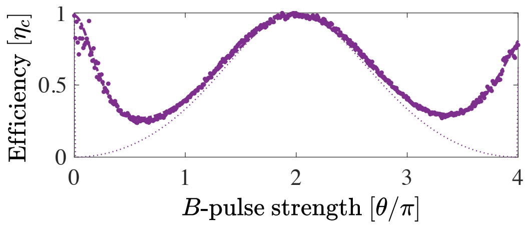

Supplementary Figure 2. Corresponding to each B-pulse strength, the coherent efficiency $\eta_c(\theta) = p_0(\theta)/[p_0(\theta) + p_2(\theta)]$ obtained from the experiment is shown as a purple line with circular markers, closely followed by the simulated curve (purple dot-dashed). The thin dotted purple line represents the respective ideal case, with no decoherence and without any experimental imperfections.

Quantizing the B-pulse in the $N = 1$ case

We can get a deeper understanding of this effect by looking at the case where we treat the B-pulse quantum mechanically rather that in the semiclassical approximation. Let us denote by b and $b^\dagger$ the annihilation and creation operator describing the presence of photons from the B-pulse. In the rotating wave approximation, the interaction Hamiltonian between the pulse and the transmon is

$$H_{\rm int} = \frac{i\hbar g}{2}\left(b^\dagger |1\rangle\langle 2| - b|2\rangle\langle 1|\right). \tag{10}$$

Consider now a Fock state $|\mathtt{n}\rangle$ with $\mathtt{n}$ photons. Experimentally, this can be realized as a cavity or resonator to which the transmon can be coupled and uncoupled. The Hamiltonian Eq. (10) conserves the number of excitations in the total Hilbert space of the resonator and the second transmon transition. As a result, the dynamics is confined to the subspace spanned by the vectors $|\mathtt{n}\rangle \otimes |1\rangle$ and $|\mathtt{n}-1\rangle \otimes |2\rangle$. In this subspace the Hamiltonian can be diagonalized; we obtain the eigenvectors

$$|\mathtt{n}+\rangle = \frac{1}{\sqrt{2}}\left(|\mathtt{n}\rangle \otimes |1\rangle - i|\mathtt{n}-1\rangle \otimes |2\rangle\right), \tag{11}$$

$$|\mathtt{n}-\rangle = \frac{-1}{\sqrt{2}}\left(|\mathtt{n}\rangle \otimes |1\rangle + i|\mathtt{n}-1\rangle \otimes |2\rangle\right), \tag{12}$$

and the eigenvalues $E_\pm = \pm(\hbar/2)g\sqrt{\mathtt{n}}$, corresponding to a Rabi frequency $g\sqrt{n}$. Assume now a certain duration of the B-pulse – let's denote it $\tau_{\rm B}$. We can define the corresponding strength $\theta_{\mathtt{n}}$ of the $\mathtt{n}$-photon pulse as $\theta_{\mathtt{n}} = g\sqrt{n}\tau_{\rm B}$. We start in the state $|\mathtt{n}\rangle \otimes |0\rangle$ and apply S_1, B (via the interaction Hamiltonian Eq.10), and again S_1. The final result is the state

$$\sin^2\frac{\theta_{\mathtt{n}}}{4}|\mathtt{n}\rangle \otimes |0\rangle + \cos^2\frac{\theta_{\mathtt{n}}}{4}|\mathtt{n}\rangle \otimes |1\rangle + \frac{1}{\sqrt{2}}\sin\frac{\theta_{\mathtt{n}}}{2}|\mathtt{n}-1\rangle \otimes |2\rangle. \tag{13}$$

The case when the cavity is not present ($g = 0$) can be obtained directly from the expression above or by a separate calculation involving only the two consecutive S_1 unitaries, yielding, as expected,

$$|\mathtt{n}\rangle \otimes |1\rangle. \tag{14}$$

First, we can immediately compare these results with the semiclassical expression Eq. (8), to check that the probabilities are the same. But most importantly, Eq. (13) shows the entanglement and the energy balance between the pulse and the detector: if the transmon is found in the state $|0\rangle$ then the pulse will still contain $\mathtt{n}$ photons, *i.e.*, no photon has been absorbed. On the contrary, if the transmon is found in state $|2\rangle$, this could happen only with the absorption of a photon from the B-pulse. In the case that state $|1\rangle$ is detected we cannot conclude anything, but we can still rest assured that the cavity is not affected even if it was present in the setup.

B. $N = 2$ case

For the $N = 2$ case the beam splitter S_2 is a $\pi/3$ pulse

$$S_2 = \frac{\sqrt{3}}{2}\mathbb{I}_{01} - \frac{i}{2}\sigma^y_{01} + |2\rangle\langle 2|. \tag{15}$$

The final state is

$$\begin{aligned}
&\frac{1}{8}\left[3\sqrt{3} - \sqrt{3}\cos\frac{\theta_1}{2} - 2\sqrt{3}\cos^2\frac{\theta_1}{4}\cos\frac{\theta_2}{2}\right.\\
&\left.\quad +2\sin\frac{\theta_1}{2}\sin\frac{\theta_2}{2}\right]|0\rangle + \frac{1}{8}\left[3 - \cos\frac{\theta_1}{2}\right.\\
&\left.+6\cos^2\frac{\theta_1}{4}\cos\frac{\theta_2}{2} - 2\sqrt{3}\sin\frac{\theta_1}{2}\sin\frac{\theta_2}{2}\right]|1\rangle\\
&+\frac{1}{2}\left[\sin\frac{\theta_1}{2}\cos\frac{\theta_2}{2} + \sqrt{3}\cos^2\frac{\theta_1}{4}\sin\frac{\theta_2}{2}\right]|2\rangle.
\end{aligned} \tag{16}$$

At maximum strength $\theta_1 = \theta_2 = \pi$ this state reads

$$\frac{1}{8}(2 + 3\sqrt{3})|0\rangle + \frac{1}{8}(3 - 2\sqrt{3})|1\rangle + \frac{1}{4}\sqrt{3}|2\rangle. \tag{17}$$

We can already see that the probability of absorption is $p_2 = 3/16 = 0.1875$, smaller than the 0.25 of the single-interrogation detection, and the probability p_0 of an IFM detection is $p_0 = (31 + 12\sqrt{3})/64 \approx 0.8091$, significantly larger than the 0.25 of the single-interrogation case. The efficiency of the coherent detection is

$$\eta_c = \frac{p_0}{p_0 + p_2} = 0.8118. \tag{18}$$

Note that the efficiency is so high because the probability of failing to find the pulse is very small, $p_1 = 0.0034$.

Further, we experimentally realize a general case where B-pulse strengths are different, *i.e.*, $\theta_1, \theta_2 \in [0, 4\pi]$. Maps of the experimental and simulated results for the efficiency η_c are shown as functions of θ_1 and θ_2 in Supplementary Fig. 3. The variation of the ground state, first excited state and second excited state probabilities as functions of θ_1 and θ_2 is shown in the main text alongside with the Positive Ratio $PR(\theta_1, \theta_2) = p_0(\theta_1, \theta_2)/(p_0(\theta_1, \theta_2) + p_1(\theta_1, \theta_2))$ and the Negative Ratio $NR(\theta_1, \theta_2) = p_1(\theta_1, \theta_2)/(p_0(\theta_1, \theta_2) + p_1(\theta_1, \theta_2))$. Experimental and simulated results are in very good agreement with each other.

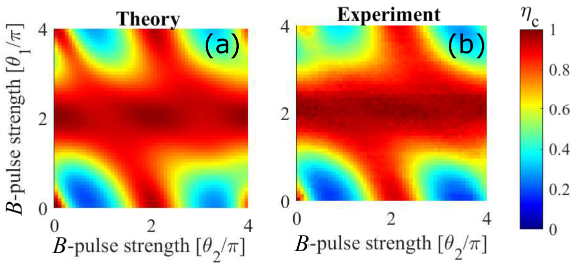

Supplementary Figure 3. Simulated and experimental map of coherent efficiency η_c as a function of θ_1 and θ_2.

Importantly, we also observe that the previous maximum at $\theta = 2\pi$ from the $N = 1$ case (see Fig. 2 in the main text) starts to flatten, evolving towards becoming a plateau, a tendency that will become even more prominent for $N \gg 1$.

Let us now clarify the difference with respect to the standard projective (non-unitary) interaction-free measurement, considering for simplicity the case $\theta_1 = \theta_2 = \pi$. After the first pulse of strength θ_1 the state becomes

$$B(\theta_1)S_2|0\rangle = \frac{\sqrt{3}}{2}|0\rangle + \frac{1}{2}\cos\frac{\theta_1}{2}|1\rangle + \frac{1}{2}\sin\frac{\theta_1}{2}|2\rangle. \tag{19}$$

This is the state that serves as the input for the next Ramsey S_2 pulse.

We can now see that there is a crucial difference with respect to the case when there has been a measurement of the second excited state and the result was negative. In this situation, the state entering the second S_2 pulse is

$$\frac{1}{\sqrt{p_{\overline{\mathrm{abs}}}(\theta_1)}} P_{\overline{\mathrm{abs}}} B(\theta_1)S_2|0\rangle = \\ = \frac{1}{\sqrt{3 + \cos^2(\theta_1/2)}} \left(\sqrt{3}|0\rangle + \cos\frac{\theta_1}{2}|1\rangle\right) \tag{20}$$

where $p_{\overline{\mathrm{abs}}}(\theta_1) = \frac{3}{4} + \frac{1}{4}\cos^2(\theta_1/2)$. Unlike Eq. (19), this state does not have a component on $|2\rangle$. In the case $\theta_1 = \pi$, the state Eq. (20) seen by the second S_2 pulse is $|0\rangle$, the same as the initial one. Thus, the same interference phenomena is reproduced in the second Ramsey cycle. In contrast, for the coherent case, Eq. (19) contains a component on $|2\rangle$, which precisely encapsulates our lack of knowledge about the p_2 probability at the beginning of the second Ramsey cycle.

We can also calculate the probabilities and efficiency in the non-unitary case for $\theta_1 = \theta_2 = \pi$, $p_{\mathrm{det}} = (\cos^2(\pi/6))^3 = 27/64 = 0.4219$ and $p_{\mathrm{abs}} = \sin^2(\pi/6)[1 + \cos^2(\pi/6)] = 7/16 = 0.4375$, resulting in an efficiency

$$\eta = \frac{p_{\mathrm{det}}}{p_{\mathrm{det}} + p_{\mathrm{abs}}} = 0.49. \tag{21}$$

We see for the case of $N = 2$ that the efficiency of the coherent case is significantly larger!

Quantizing the B-pulse in the N = 2 case

In a similar way to the $N = 1$ case, we can treat the B-pulse quantum mechanically. We consider that an interaction Hamiltonian Eq. (10) is available, such that the transmon can be coupled in a controllable way to the field.

Suppose that the transmon is coupled in both sequences to the same mode containing $\mathtt{n}$ photons. These photons can be for example located in a cavity, which is coupled by a tunable coupling element to the transmon, or they can be traveling in a transmission line, as in our experiments. The initial state is $|\mathtt{n}\rangle \otimes |0\rangle$. The final state can be obtained by the same procedure as in the $N = 1$ case, and reads

$$\frac{1}{8}\left[3\sqrt{3} - \sqrt{3}\cos\frac{\theta_{1\mathtt{n}}}{2} - 2\sqrt{3}\cos^2\frac{\theta_{1\mathtt{n}}}{4}\cos\frac{\theta_{2\mathtt{n}}}{2} + \\ +2\sin\frac{\theta_{1\mathtt{n}}}{2}\sin\frac{\theta_{2\mathtt{n}}}{2}\right]|\mathtt{n}\rangle\otimes|0\rangle + \\ \frac{1}{8}\left[3 - \cos\frac{\theta_{1\mathtt{n}}}{2} + 6\cos^2\frac{\theta_{1\mathtt{n}}}{4}\cos\frac{\theta_{2\mathtt{n}}}{2} \\ -2\sqrt{3}\sin\frac{\theta_{1\mathtt{n}}}{2}\sin\frac{\theta_{2\mathtt{n}}}{2}\right]|\mathtt{n}\rangle\otimes|1\rangle + \\ +\frac{1}{2}\left[\sin\frac{\theta_{1\mathtt{n}}}{2}\cos\frac{\theta_{2\mathtt{n}}}{2} + \sqrt{3}\cos^2\frac{\theta_{1\mathtt{n}}}{4}\sin\frac{\theta_{2\mathtt{n}}}{2}\right]|\mathtt{n}-1\rangle\otimes|2\rangle \tag{22}$$

with the notation $\theta_{1\mathtt{n}} = g\sqrt{n}t_{\mathrm{B1}}$ and $\theta_{2\mathtt{n}} = g\sqrt{n}t_{\mathrm{B2}}$. We immediately observe the similarity with the semiclassical

result Eq. (16). The result very clearly reaffirms that the photonic Fock state does not change by finding the qubit in the state $|0\rangle$. It can lose a photon only if the level $|2\rangle$ is excited. Thus, we can detect the existence of photons inside the cavity without absorbing any of them.

Generalization to two different modes: We can also imagine the situation when the transmon is coupled to different modes in the two sequences, for example realized as photons in two distinct cavities. Suppose that in the first sequence it interacts with a cavity containing $\mathtt{n}$ photons, while in the second sequence it interacts with another cavity, containing $\mathtt{n}$ photons. The initial state is then $|\mathtt{m},\mathtt{n}\rangle \otimes |0\rangle$. The final state in this case can be calculated as

$$\begin{aligned}
&\frac{1}{8}\left[\left(3\sqrt{3}-\sqrt{3}\cos\frac{\theta_{1\mathtt{n}}}{2}-2\sqrt{3}\cos^2\frac{\theta_{1\mathtt{n}}}{4}\cos\frac{\theta_{2\mathtt{m}}}{2}\right)|\mathtt{m},\mathtt{n}\rangle\right.\\
&\left.+2\sin\frac{\theta_{1\mathtt{n}}}{2}\sin\frac{\theta_{2\mathtt{m}+1}}{2}|\mathtt{m}+1,\mathtt{n}-1\rangle\right]\otimes|0\rangle+\\
&\frac{1}{8}\left[\left(3-\cos\frac{\theta_{1\mathtt{n}}}{2}+6\cos^2\frac{\theta_{1\mathtt{n}}}{4}\cos\frac{\theta_{2\mathtt{m}}}{2}\right)|\mathtt{m},\mathtt{n}\rangle\right.\\
&\left.-2\sqrt{3}\sin\frac{\theta_{1\mathtt{n}}}{2}\sin\frac{\theta_{2\mathtt{m}+1}}{2}|\mathtt{m}+1,\mathtt{n}-1\rangle\right]\otimes|1\rangle\\
&+\frac{1}{2}\left[\sin\frac{\theta_{1\mathtt{n}}}{2}\cos\frac{\theta_{2\mathtt{m}+1}}{2}|\mathtt{m},\mathtt{n}-1\rangle+\right.\\
&\left.\sqrt{3}\cos^2\frac{\theta_{1\mathtt{n}}}{4}\sin\frac{\theta_{2\mathtt{m}}}{2}|\mathtt{m}-1,\mathtt{n}\rangle\right]\otimes|2\rangle, \qquad (23)
\end{aligned}$$

with the notation $\theta_{1\mathtt{n}} = g_1\sqrt{n}t_{\mathrm{B1}}$ and $\theta_{2\mathtt{m}} = g_2\sqrt{m}t_{\mathrm{B2}}$, $\theta_{2\mathtt{m}+1} = g_2\sqrt{m+1}t_{\mathrm{B2}}$. If the transmon gets excited, we see that this can happen with the loss of a photon from either one of the modes. If the transmon is found in the state $|0\rangle$, then we can ascertain the existence of photons in the cavities, and, at the same time, we have transformed the initial Fock state $|\mathtt{m},\mathtt{n}\rangle$ into a coherent superposition of $|\mathtt{m},\mathtt{n}\rangle$ and $|\mathtt{m}+1,\mathtt{n}-1\rangle$. The latter of course represents the possibility that a photon gets absorbed by the transmon during the first Ramsey sequence and reemitted into the same cavity during the second sequence. The transformation of a Fock state into a coherent state is a feature that is reminiscent of the famous Hanbury Brown-Twiss experiment [4].

C. $N > 1$ case

We have seen that for $N = 1$ the efficiency of the coherent protocol is the same as that of the projective protocol, while for $N = 2$ the coherent protocol is more advantageous. Does this tendency continues for large N? Let us take one more step and look at the case $N = 3$. In the coherent protocol, the output state is

$$\begin{aligned}
&\cos\frac{\pi}{8}\left(\cos^3\frac{\pi}{8}+2\sin^2\frac{\pi}{8}\right)|0\rangle\\
&+\sin\frac{\pi}{8}\left(\cos^3\frac{\pi}{8}+\sin^2\frac{\pi}{8}-\cos^2\frac{\pi}{8}\right)|1\rangle\\
&+\sin\frac{\pi}{8}\cos\frac{\pi}{8}\left(\cos\frac{\pi}{8}-1\right)|2\rangle. \qquad (24)
\end{aligned}$$

We can verify immediately that $p_0 > \cos^8(\pi/8) = p_{\mathrm{det}}$ and $p_2 < \sin^2(\pi/8)(1+\cos^2(\pi/8)+\cos^4(\pi/8)) = p_{\mathrm{abs}}$, where p_{det} and p_{abs} are the detection and absorption probabilities in the $N = 3$ case, respectively.

We can now generalize the protocol to N B-pulses and the same number of Ramsey sequences. In this case the S pulses are defined as

$$S_N = \exp\left[-i\frac{\pi}{2(N+1)}\sigma_{01}^y\right]. \qquad (25)$$

The efficiency of the coherent detection is defined as before:

$$\eta_c = \frac{p_0}{p_0+p_2}. \qquad (26)$$

Let us now consider the non-unitary (projective) protocol. In this case the probability of a successful detection is the product of probabilities that the system stays in the state $|0\rangle$

$$p_{\mathrm{det}} = \left[\cos\left(\frac{\pi}{2(N+1)}\right)\right]^{2(N+1)}, \qquad (27)$$

while the absorption probability is

$$p_{\mathrm{abs}} = \sin^2\left(\frac{\pi}{2(N+1)}\right)\sum_{n=0}^{N-1}\left[\cos\left(\frac{\pi}{2(N+1)}\right)\right]^{2n}. \qquad (28)$$

Note that $[\cos(\pi/2(N+1))]^{2(N+1)} \overset{N\gg1}{\approx} 1-\pi^2/4(N+1)+\mathcal{O}[N^{-2}]$, therefore p_{det} approaches unity at large N. The second expression is a sum of independent probabilities (that there is absorption in the first Ramsey sequence, that there is no absorption in the first Ramsey sequence but there is in the second, etc.). The efficiency is

$$\eta = \frac{p_{\mathrm{det}}}{p_{\mathrm{det}}+p_{\mathrm{abs}}}. \qquad (29)$$

The efficiencies obtained in the coherent and the projective cases for N$\in[1,25]$ are plotted in Supplementary Fig. 4, with and without decoherence. Clearly, the efficiency obtained in the coherent case is significantly higher than that of the projective case, *i.e.* $\eta_c > \eta$ and $\eta_c^{(d)} > \eta^{(d)}$ for any value of $N > 1$. In the presence of decoherence, the difference between the two cases tends to stay constant with increasing N.

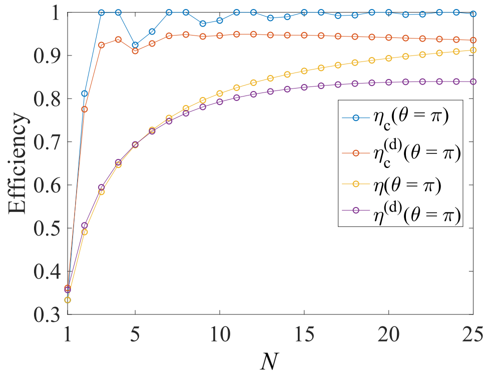

Supplementary Figure 4. The efficiency of our scheme η_c as a function of N Ramsey segments is compared with the efficiency of the standard projective scheme η, all at strength $\theta = \pi$. The corresponding cases with dissipation included are denoted by $\eta_c^{(d)}$ and $\eta^{(d)}$.

Elements of the confusion matrix in coherently repeated interrogations. We can obtain the elements of the confusion matrix in a more general form for the case of N B-pulses, see Supplementary Fig. (5). The general 2D maps of these positive and negative ratios are plotted as functions of the number of B-pulses and B-pulse strength as shown in Supplementary Fig. (5) for ideal simulation without decoherence, simulation with decoherence, and results from the experiments. It is clear from the surface maps in Supplementary Fig. (5) that the True Positive Ratio (TPR) is close to 1 and the False Negative Ratio (FNR) is close to 0 for $N > 2$ as observed from the simulated and the experimental results. Ideally, FPR and TNR are independent of N, but there is an increase in FPR values and a decrease in TNR values with increasing N in parts (b1,b2,c1,c2) of Supplementary Fig. (5), which is due to the long sequences, where decoherence is significant. The experimental data used in this section for arbitrary N correspond to the case of equal B-pulse strengths varying linearly between $[0, \pi]$; consistent with the data shown in Fig. 6(a) of the main text.

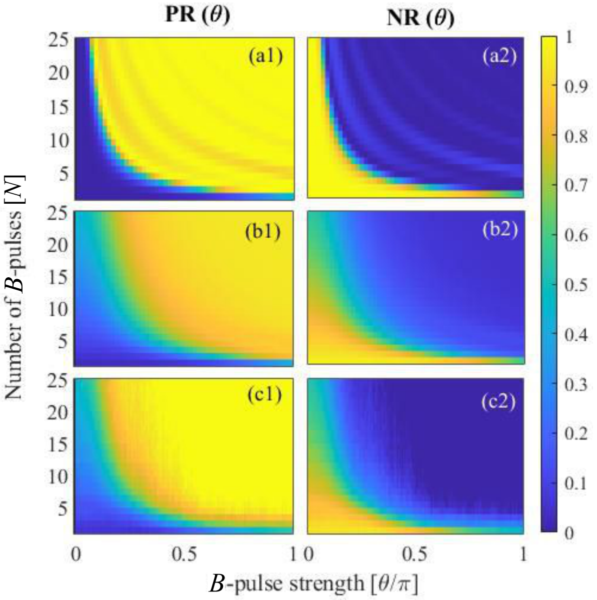

Supplementary Figure 5. (a1, b1, c1) Positive ratio, PR (θ) and (a2, b2, c2) Negative ratio, NR (θ) are plotted for different number of B-pulses N with equal strengths θ, where $\theta \in [0, \pi]$ linearly sweeps the range of B-pulse strengths for a given $N \in [1, 25]$. Panels (a1,a2) present the ideal situation with no decoherence, panels (b1,b2) are the results obtained from simulation with decoherence and panels (c1,c2) correspond to the results from the experiments.

We also obtain the coherent interaction-free efficiency η_c as a function of the B-pulse equal-strength θ and number of B-pulses N. The simulated values of η_c are shown as a surface plot in Supplementary Fig. 6(a) with a few experimental values for various combinations of (N, θ) marked on top of the surface plot. The continuous black curve corresponds to the simulated values of $\eta_c = 0.85$. Supplementary Fig. 6(b) shows the simulated (continuous line) and experimental values (black circular markers) of η_c at maximum B-pulse strength $\theta = \pi$ at various N's. Clearly, the simulation and corresponding experimental values depict a wide region of highly efficient interaction-free detection of the B-pulses.

Next, let us reconsider the p_0 profiles for various different values of N as a function of θ. As expected, p_0 gradually rises from 0 to a maximum value with increasing θ and then tends to stay higher, forming a plateau which is symmetrical around $\theta = 2\pi$. This plateau gets wider with increasing N. We quantify the widening in terms of the area $\int_0^{\pi} d\theta p_0(\theta)$ enclosed under p_0 – as a function of θ – for a given N and for $\theta \in [0, \pi]$. The results for equal and unequal arbitrary B-pulse strengths are shown in Supplementary Fig. 7 (a,b) respectively. Evaluation of area from the experimental data is shown with red square markers, with the simulation as continuous black curve. The dotted black curve is the simulation without considering the depolarization channel, while the dashed black curve signifies the ideal case without decoherence. As expected, the simulation in the absence of depolarization predicts higher values than without depolarization, while the ideal case provides the upper limit to the area. Note also that the respective plots of area for unequal B-pulse strengths are higher than for equal B-pulse strengths. Once again this conveys the idea that unequal random B-pulse strengths give rise to higher efficiency of coherent interaction-free detection.

Another important observation is that, in order to work properly, the protocol should start with the transmon in state $|0\rangle$. This is because the imbalance in the beam-splitter is designed such that the B-pulse is probed only weakly at each pass, with most of the weight of the superposition meant to stay in the $|0\rangle$ state. This, of course, is also the case for the optical projective realizations. To understand this better, we can simulate the situation where we start in state $|1\rangle$ for the case of uni-

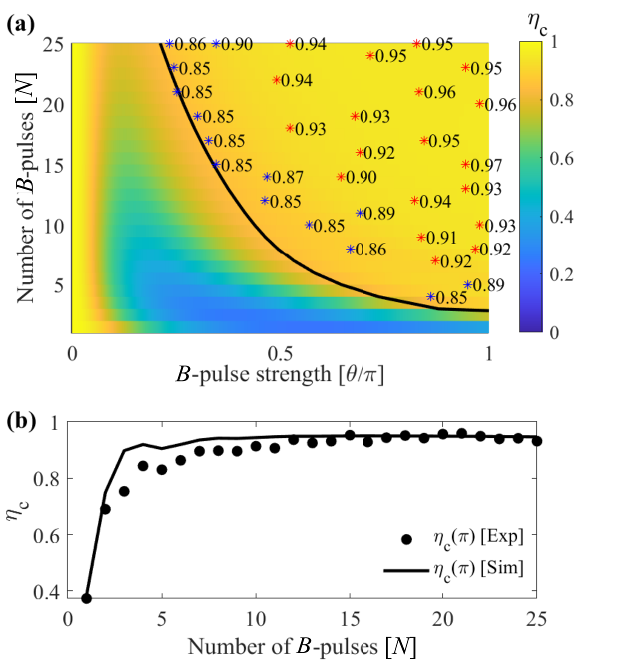

Supplementary Figure 6. (a) Surface plot of simulated η_c, plotted versus the number of B-pulses and θ with explicit experimental values marked on top of it. Blue markers correspond to experimental η_c in the range $[0.85, 0.90)$ and red markers correspond to experimental $\eta_c > 0.90$. The continuous black curve corresponds to simulated $\eta_c = 0.85$. The formation of a plateau of high efficiency values is thus confirmed experimentally. (b) Coherent interaction-free efficiency is plotted as a function of N for $\theta = \pi$, where the continuous back curve presents the simulated profile $\eta_c(\theta = \pi)$ and the circular markers correspond to the respective experimental values.

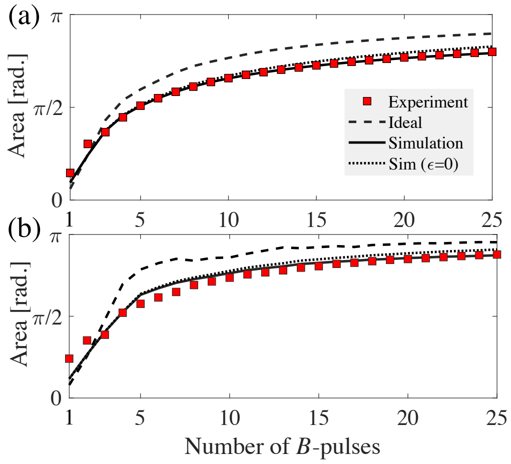

Supplementary Figure 7. Area under the $p_0(\theta)$ plots as a function of N with $\theta \in [0, \pi]$ for (a) Identical B-pulses with linearly varying strengths, (b) Randomly chosen B-pulses.

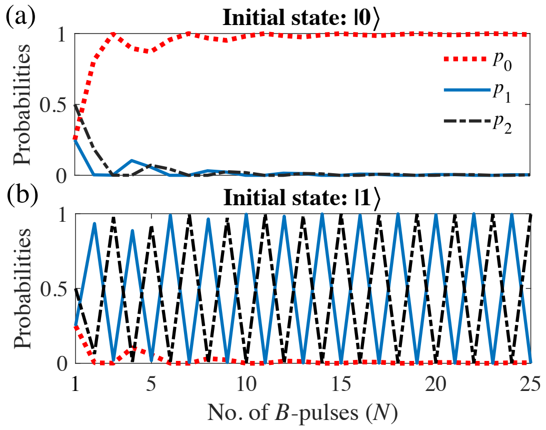

Supplementary Figure 8. Probabilities at various N for $\theta = \pi$, with initial transmon state (a) $|0\rangle$ and (b) $|1\rangle$.

form values $\theta = \pi$, see Supplementary Fig. 8. One can see that if the protocol is run correctly, with the ground state as the initial state, the probabilities stabilize relatively fast to the values $p_0 \approx 1$, $p_1 \approx p_2 \approx 0$. But in the case when we start with $|1\rangle$, the $\theta = \pi$ excitation is shuffled between the transmon and the pulse and the protocol does not yield some stationary values. Indeed, the state after each odd pulse N leaves the transmon in the state $|2\rangle$ so nothing happens at the $N+1$ beam-splitter. Then, at the next encounter with the pulse (even $N+1$) the transmon goes in the state $|1\rangle$ by stimulated emission. As it encounter the $N+2$ beam-splitter, the transmon remains mostly on the state $|1\rangle$ due to the asymmetry of the beam-splitter. Then it sees again an odd $N+2$ pulse, etc.

Quantizing the B-pulse in the $N > 1$ case

Similarly to $N = 2$, if we interrogate a single mode with $\mathtt{n}$ photons, the 3x3 matrix structure of the semiclassical case will be preserved with the replacement $|0\rangle \to |\mathtt{n}\rangle \otimes |0\rangle$, $|1\rangle \to |\mathtt{n}\rangle \otimes |1\rangle$, and $|2\rangle \to |\mathtt{n}-1\rangle \otimes |2\rangle$, as it is clear from the $N = 2$ case already. Thus, all the results obtained in this paper can be applied to this situation as well.

We can get further insights into the nature of the measurement by examining a toy-model where instead of a cavity we have a two-level system with energy levels $|\mathtt{0}\rangle$ and $|\mathtt{1}\rangle$ that can resonantly exchange energy with the second transition of the transmon. Suppose now that we apply our protocol with a large enough $N \gg 1$, with the qubit initially in a generic superposition $\alpha|\mathtt{0}\rangle + \beta|\mathtt{1}\rangle$. Based on our result so far, we would have

$$(\alpha|\mathtt{0}\rangle + \beta|\mathtt{1}\rangle) \otimes |0\rangle \xrightarrow{N\gg 1} \alpha|\mathtt{0}\rangle \otimes |1\rangle + \beta|\mathtt{1}\rangle \otimes |0\rangle. \quad (30)$$

This looks very similar to a CNOT gate with the qubit

as the control followed by an X gate on the target,

$$\begin{aligned}(\alpha|0\rangle + \beta|1\rangle) \otimes |0\rangle &\xrightarrow{CNOT} \alpha|0\rangle \otimes |0\rangle + \beta|1\rangle \otimes |1\rangle \\ &\xrightarrow{X} \alpha|0\rangle \otimes |1\rangle + \beta|1\rangle \otimes |0\rangle\end{aligned} \tag{31}$$

But the similarity stops here. Indeed, the CNOT and the X gate act on the rest of the states as

$$\begin{aligned}(\alpha|0\rangle + \beta|1\rangle) \otimes |1\rangle &\xrightarrow{CNOT} \alpha|0\rangle \otimes |1\rangle + \beta|1\rangle \otimes |0\rangle \\ &\xrightarrow{X} \alpha|0\rangle \otimes |0\rangle + \beta|1\rangle \otimes |1\rangle\end{aligned} \tag{32}$$

as usual. However, when using our protocol we have the following action on these states

$$(\alpha|0\rangle + \beta|1\rangle) \otimes |1\rangle \xrightarrow[N\text{odd}]{N\gg 1} -\alpha|0\rangle \otimes |0\rangle - i^{N+1}\beta|0\rangle \otimes |2\rangle, \tag{33}$$

and

$$(\alpha|0\rangle + \beta|1\rangle) \otimes |1\rangle \xrightarrow[N\text{even}]{N\gg 1} -\alpha|0\rangle \otimes |0\rangle + i^{N}\beta|1\rangle \otimes |1\rangle, \tag{34}$$

The action $|0\rangle \otimes |1\rangle \rightarrow -|0\rangle \otimes |0\rangle$ results immediately from $[S_N]^{N+1} = -i\sigma^y_{01} + |2\rangle\langle 2|$, implying $\mathbb{I}_2 \otimes [S_N]^{N+1}|0\rangle \otimes |1\rangle = -|0\rangle \otimes |0\rangle$. The operations $|1\rangle \otimes |1\rangle \xrightarrow[N\text{odd}]{N\gg 1} |0\rangle \otimes |2\rangle$ and $|1\rangle \otimes |1\rangle \xrightarrow[N\text{even}]{N\gg 1} |1\rangle \otimes |1\rangle$ can be verified numerically, see for example Supplementary Fig. 8 as well as the approximate formula for the unitary at large N given in the Discussion section in the main paper. Again this has a straightforward physical interpretation: after the application of an even number N of pulses (that is, immediately before the $N+1$ beam-splitter), the state of the system is approximately $|1\rangle \otimes |1\rangle$, that is, the qubit is excited and the transmon is in state $|1\rangle$. Since N is large, after acting with the $N+1$ beam-splitter the transmon still remains approximately in the state $|1\rangle$: it can then fully absorb the excitation at the $N+1$ interaction with the qubit. This results in the state $|0\rangle \otimes |2\rangle$. Further on, nothing happens at the $N+2$ beam-splitter, since this acts only on the states $|0\rangle$ and $|1\rangle$. Then the $N+2$ interaction with the qubit will result in the excitation being transferred from $|2\rangle$ to the qubit. As a result, before the $N+3$ beam-splitter the state will be $|1\rangle \otimes |1\rangle$, which is exactly the state it entered the $N+1$ beam-splitter. The whole process then just repeats.

This shows that our protocol is fundamentally different from the standard von Neumann measurement model, which in its simplest formulation uses a CNOT to entangle the control qubit and the target meter. Perhaps even more relevant for our problem, it is not even possible to construct a CNOT gate based only on the Hamiltonian Eq. (10), which would generate just an iSWAP type of gate. To construct a CNOT, one would need additional single-qubit gates for both the target and control [5], meaning that additional energy is exchanged, see e.g. Ref. [6] for an explicit construction in an experiment on measuring the state of a nuclear spin.

Generalization to multiple modes. A different scenario can be envisioned if several different modes are available, when clearly a variety of options exist on how to interrogate them. In this case, states that correspond to superpositions of these modes will be obtained when the transmon is found in the ground state, similar to what has already been observed for $N = 2$. Thus, our protocol can be generalized to simultaneous detection of photons in several cavities.

SUPPLEMENTARY NOTE 3: GENERAL RESULTS FOR $N \geq 1$ COHERENT INTERACTION-FREE DETECTION

A number of theoretical results for the case of $N \geq 1$ are presented in this section.

General analytical results

For the coherent case, the subsequent evolution for a system of size N is just $(SB)^N S|0\rangle$. Let us denote the wavefunction after the jth Ramsey segment as $|\psi\rangle_j = \alpha_j|0\rangle + \beta_j|1\rangle + \gamma_j|2\rangle$. The probability amplitudes $\alpha_j, \beta_j, \gamma_j$ obey the recursion relations

$$\begin{aligned}\alpha_{j+1} &= \cos\frac{\pi}{2(N+1)}\alpha_j \qquad (35)\\ &\quad - \sin\frac{\pi}{2(N+1)}\cos\frac{\theta_{j+1}}{2}\beta_j + \sin\frac{\pi}{2(N+1)}\sin\frac{\theta_{j+1}}{2}\gamma_j, \\ \beta_{j+1} &= \sin\frac{\pi}{2(N+1)}\alpha_j \qquad (36)\\ &\quad + \cos\frac{\pi}{2(N+1)}\cos\frac{\theta_{j+1}}{2}\beta_j - \cos\frac{\pi}{2(N+1)}\sin\frac{\theta_{j+1}}{2}\gamma_j, \\ \gamma_{j+1} &= \sin\frac{\theta_{j+1}}{2}\beta_j + \cos\frac{\theta_{j+1}}{2}\gamma_j \,. \qquad (37)\end{aligned}$$

In the case of identical pulses $\theta_j = \theta$, starting with the probability amplitudes Eqs. (35, 36, 37), we observe that these recursion relations yield sums of even functions of θ (cosines) α_j and β_j, and sums of odd functions of θ (sines) γ_j. Specifically, the amplitudes in the coherent case can be expressed as the expansions

$$\alpha_j = \sum_{k=0}^{j} C_j[k]\cos\frac{k\theta}{2}, \tag{38}$$

$$\beta_j = \sum_{k=0}^{j} C'_j[k]\cos\frac{k\theta}{2}, \tag{39}$$

$$\gamma_j = \sum_{k=0}^{j} C''_j[k]\sin\frac{k\theta}{2}. \tag{40}$$

From the recursion relations Eqs. (35, 36, 37), we find

the following relations among the coefficients

$$C_{j+1}[k] = \cos\frac{\pi}{2(N+1)}C_j[k]_{k=\overline{0,j}} - \frac{1}{2}\sin\frac{\pi}{2(N+1)}\left[C'_j[k+1] - C''_j[k+1]\right]_{k=\overline{-1,j-1}} - \frac{1}{2}\sin\frac{\pi}{2(N+1)}\left[C'_j[k-1] + C''_j[k-1]\right]_{k=\overline{1,j+1}} \quad (41)$$

$$C'_{j+1}[k] = \sin\frac{\pi}{2(N+1)}C_j[k]_{k=\overline{0,j}} + \frac{1}{2}\cos\frac{\pi}{2(N+1)}\left[C'_j[k+1] - C''_j[k+1]\right]_{k=\overline{-1,j-1}} + \frac{1}{2}\cos\frac{\pi}{2(N+1)}\left[C'_j[k-1] + C''_j[k-1]\right]_{k=\overline{1,j+1}} \quad (42)$$

$$C''_{j+1}[k] = \frac{1}{2}\left[C'_j[k-1] + C''_j[k-1]\right]_{k=\overline{1,j+1}} - \frac{1}{2}\left[C'_j[k+1] - C''_j[k+1]\right]_{k=\overline{-1,j}} \quad (43)$$

Here we use the notation $[...]_{k=\overline{..,..}}$ to denote a restriction over the values of k. The final probabilities can then be easily calculated as $p_0 = \alpha_N^2$, $p_1 = \beta_N^2$, and $p_2 = \gamma_N^2$. The coefficients for systems of sizes $N = 1$, $N = 2$, $N = 3$, and $N = 4$ are shown in Supplementary Tables. II–IV.

	$N=1$	$N=2$	$N=3$	$N=4$
$\mathbf{C}_N[\mathbf{0}]$	0.5	0.67	0.75	0.80
$\mathbf{C}_N[\mathbf{1}]$	−0.5	−0.43	−0.36	−0.31
$\mathbf{C}_N[\mathbf{2}]$	0	−0.23	−0.25	−0.24
$\mathbf{C}_N[\mathbf{3}]$	0	0	−0.14	−0.16
$\mathbf{C}_N[\mathbf{4}]$	0	0	0	−0.089

Supplementary Table II. The probability amplitude coefficients for p_0 (coherent case) up to two significant digits with sequences of length $N = 1$, $N = 2$, $N = 3$, and $N = 4$.

	$N=1$	$N=2$	$N=3$	$N=4$
$\mathbf{C}'_N[\mathbf{0}]$	0.5	0.35	0.27	0.22
$\mathbf{C}'_N[\mathbf{1}]$	0.5	0.25	0.17	0.13
$\mathbf{C}'_N[\mathbf{2}]$	0	0.40	0.23	0.17
$\mathbf{C}'_N[\mathbf{3}]$	0	0	0.33	0.21
$\mathbf{C}'_N[\mathbf{4}]$	0	0	0	0.27

Supplementary Table III. The probability amplitude coefficients for p_1 (coherent case) up to two significant digits with sequences of length $N = 1$, $N = 2$, $N = 3$, and $N = 4$.

	$N=1$	$N=2$	$N=3$	$N=4$
$\mathbf{C}''_N[\mathbf{1}]$	0.71	0.43	0.33	0.27
$\mathbf{C}''_N[\mathbf{2}]$	0	0.47	0.31	0.24
$\mathbf{C}''_N[\mathbf{3}]$	0	0	0.35	0.25
$\mathbf{C}''_N[\mathbf{4}]$	0	0	0	0.29

Supplementary Table IV. The probability amplitude coefficients for p_2 (coherent case) up to two significant digits with sequences of length $N = 1$, $N = 2$, $N = 3$, and $N = 4$.

The recurrence relations allow us to get a deeper understanding of the process of coherent accumulation of amplitude probabilities in successive pulses. Let us consider the maximum-strength pulses $\theta_j = \pi$, for which the relations Eqs. (35, 36, 37) become

$$\alpha_{j+1} = \cos\frac{\pi}{2(N+1)}\alpha_j + \sin\frac{\pi}{2(N+1)}\gamma_j, \quad (44)$$

$$\beta_{j+1} = \sin\frac{\pi}{2(N+1)}\alpha_j - \cos\frac{\pi}{2(N+1)}\gamma_j, \quad (45)$$

$$\gamma_{j+1} = \beta_j. \quad (46)$$

We notice that if the dominant probability amplitude is the one corresponding to the ground state, this relationship tends to be preserved under successive application of the sequences. Indeed, from Eq. (46) we see that if β_j is small, then γ_{j+1} will be small as well. From Eq. (45) we see that the relatively large probability amplitude α_j gets multiplied by a small number $\sin\frac{\pi}{2(N+1)}$, and the remaining part of the equation also contains the relatively small γ_j. To make this observation more precise, we note that the general form of the probability amplitudes is

$$\alpha_j = \cos^{j+1}\frac{\pi}{2(N+1)} + \sin^2\frac{\pi}{2(N+1)}\mathcal{P}^{(j-2)}_{\alpha_j}\left(\cos\frac{\pi}{2(N+1)}\right),$$

$$\beta_j = \sin\frac{\pi}{2(N+1)}\mathcal{P}^{(j)}_{\beta_j}\left(\cos\frac{\pi}{2(N+1)}\right),$$

$$\gamma_j = \sin\frac{\pi}{2(N+1)}\mathcal{P}^{(j-1)}_{\gamma_j}\left(\cos\frac{\pi}{2(N+1)}\right).$$

where $\mathcal{P}^{(j)}$ are j-th order polynomials in the variable $\xi = \cos\frac{\pi}{2(N+1)}$ satisfying

$$\mathcal{P}^{(j-1)}_{\alpha_{j+1}}(\xi) = \xi\mathcal{P}^{(j-2)}_{\alpha_j}(\xi) + \mathcal{P}^{(j-1)}_{\gamma_j}(\xi),$$

$$\mathcal{P}^{(j+1)}_{\beta_{j+1}}(\xi) = \xi^{j+1} - \xi^2\mathcal{P}^{(j-2)}_{\alpha_j}(\xi) - \xi\mathcal{P}^{(j-1)}_{\gamma_j}(\xi) + \mathcal{P}^{(j-2)}_{\alpha_j}(\xi),$$

$$\mathcal{P}^{(j)}_{\gamma_{j+1}}(\xi) = \mathcal{P}^{(j)}_{\beta_j}(\xi).$$

We can see that the coefficients β_j and γ_j get multiplied by the small quantity $\sin\frac{\pi}{2(N+1)}$ at every iteration, therefore they tend to decrease. On the contrary, α_j accumulates the relatively larger quantity $\cos^{j+1}\frac{\pi}{2(N+1)}$, with $\lim_{N\to\infty}\cos^{N+1}\frac{\pi}{2(N+1)} = 1$. Thus, at the end of the protocol, we will have $p_0 = |\alpha_{N+1}|^2 = \cos^{2(N+1)}\frac{\pi}{2(N+1)} +$

$\sin^2 \frac{\pi}{2(N+1)}$..... The first term equals the projective probability, see Eq. (27), while the rest of terms are the result of coherent accumulation of amplitude probabilities during the sequences. We therefore expect a higher p_0 in the coherent case, and therefore a lower probability of absorption p_2. This is also calculated numerically in the next subsection.

Numerical results: cumulative probability of absorption

We have seen that the projective case of interaction-free detection completely excludes the situations where a B-pulse is absorbed by collapsing the wavefuction onto the state $|0\rangle$, which does not interact with the pulse. On the other hand, the coherent-interrogation interaction-free measurement protocol yields detection with very high probability, which is demonstrated by simulations as well as by experiments. We can introduce a figure of merit that allows us to quantify in a single number the probabilities of B-pulse absorption at different sequences. We can quantify this concisely by keeping track of the probability of absorption instances with $\theta = \pi$ at each sequence $j \in [1, N]$. For a given N we introduce $\mathbb{C} = \sum_{j=1}^{N} p_2(j)$, which essentially quantifies cumulatively the unfavorable absorption events. In Supplementary Fig. 9, the black curve corresponds to the cumulative probability with which photons can get absorbed in a projective measurement protocol and the blue curve corresponds to the total probability obtained by adding the state-$|2\rangle$ probabilities at the end of each B-pulse in the coherent measurement protocol. It is clearly seen that the coherent measurement protocol has less cumulative net probability of B-pulse absorption.

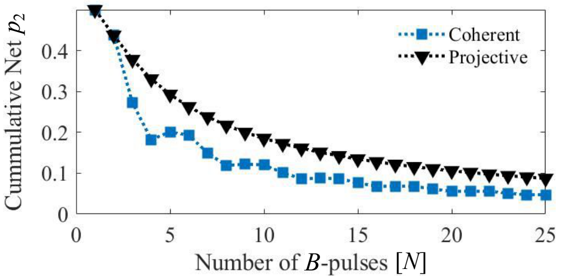

Supplementary Figure 9. Cumulative probability $\mathbb{C}$ of B-pulse absorption with $\theta = \pi$ versus N. The blue dotted curve with square markers corresponds to the case of the coherent interaction-free measurement protocol and the black dotted curve with triangular markers results from the projective measurement protocol.

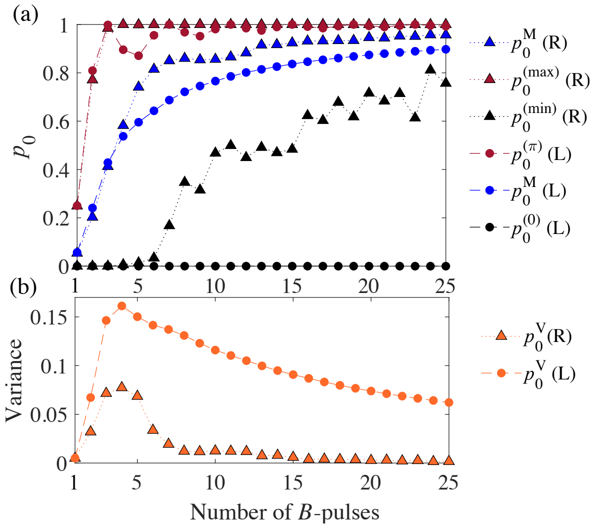

Supplementary Figure 10. (a) Mean and extreme values of p_0 are plotted for $N \in [1, 25]$ for the cases of identical B-pulses ($\theta_i = \theta_j = \theta\, i, j \in [1, N]$) as θ varies linearly from 0 to π for each N with circular markers. Triangular markers present the corresponding results when $\theta_i \neq \theta_j$ and each θ_i is chosen arbitrarily from $[0, \pi]$ independent of any θ_j. p_0^{M}(L), $p_0^{(0)}$(L), and $p_0^{(\pi)}$(L) are mean, minimum and maximum values respectively of the p_0 distribution for the case of identical B-pulses with linearly vaying strengths. p_0^{M}(R), $p_0^{(\min)}$(R), and $p_0^{(\max)}$(R) are mean, worst and best values respectively from the p_0 distribution for the case of random B-pulse strengths. (b) Curves showing the corresponding variance of the p_0 distributions with N.

Identical and random pulses

Let us have a closer look at the simulation in the case of N B-pulses with equal and unequal (random) pulse strengths. In Supplementary Fig. 10, circular markers present the case of $N \in [1, 25]$ B-pulses with equal strengths and triangular markers correspond to randomly chosen B-pulse strengths. In this case, the strengths θ of each B-pulse increase linearly from 0 to π in 400 steps and the resulting distributions of the ground state probability p_0 is obtained. As expected, in Supplementary Fig. 10(a) the black circles connected by the dashed black line representing the case of no B-pulses yields $p_0 = 0$, while the dashed line with red circular markers corresponds to $\theta = \pi$, which has a tendency to stay closer to 1. We note that $\theta = \pi$ may not correspond to the maximum value of p_0, especially for smaller values of N. In fact the maximum p_0 in both cases (equal and unequal B-pulse strengths) coincide with each other and is represented by red triangles. The average value of ground state probability p_0^{M}(L) in the case of linearly varying θ gradually increases from 0.057 for $N = 1$ to 0.897 for $N = 25$ as shown with the blue dashed curve with circles. Interestingly, the situation with randomly chosen $\theta \in [0, \pi]$ (400 samples for each N), gives rise to higher average

values ($p_0^{\mathrm{M}}(\mathrm{R})$) as shown with the blue dotted curve with triangular markers. Black and red dotted curves with triangular markers result from the worst and best combinations of random B-pulses. It is noteworthy that even the worst choice of random B-pulses have a good chance of being detected. While the ignorance about the B-pulse strengths appear to benefit in this case, results from randomly chosen B-pulse strengths also depend upon the sample size (here the sample size is 400). Further, variance of the p_0 distributions for each N is shown in Supplementary Fig. 10(b), where circular markers correspond to the case of equal B-pulse strengths and triangular markers correspond to the case of random B-pulse strengths. Much lower values of variance are obtained in the case of arbitrarily chosen B-pulse strengths.

SUPPLEMENTARY NOTE 4: DISCUSSION: IGNORANCE IS BLISS

The previous numerical simulations demonstrate that the coherent case is more efficient than the standard projective (quantum Zeno effect) case. This is a non-intuitive result, because negative measurements, while not producing any macroscopic event (detector click, etc.) still provide more information. A famous example outside quantum physics is the Monty Hall problem.

However, the strategy of extracting "classical" information is not necessarily advantageous, as the case of coherent interaction-free detection realized in this paper demonstrates. To give a qualitative justification of why it is so, let us consider the state $\sqrt{1-x^2-y^2}|0\rangle + x|1\rangle + y|2\rangle$ at the input of a Ramsey segment containing the pulse $B(\pi)$. After going through the interferometer the probability p_2 of the state $|2\rangle$ is $\left[\sqrt{1-x^2-y^2}\sin(\pi/2(N+1)) + x\cos(\pi/2(N+1))\right]^2$. Let's examine now the projective scenario. In this case, the input state should not contain any component on the state $|2\rangle$, since in this protocol the state is always projected on the $\{|0\rangle,|1\rangle\}$ subspace. Considering $\sqrt{1-x^2}|0\rangle + x|1\rangle$ as the input state, we find that the probability of detection ("explosion") is $\left[\sqrt{1-x^2}\sin(\pi/2(N+1)) + x\cos(\pi/2(N+1))\right]^2$, clearly larger than in the coherent case.

SUPPLEMENTARY NOTE 5: EXPERIMENTAL ERRORS DUE TO PULSE IMPERFECTIONS

We present here an analysis of errors due to the imperfect generation of pulses in our setup. These imperfections are: IQ mixer saturation, finite sampling rates, detunings with respect to the corresponding transition frequencies, etc. For example, IQ mixer saturation effects start to be observable in our setup for values $\theta > 3\pi$ (approximately); at the highest power, a pulse with amplitude $\Omega_0(4\pi)$ in fact implements a unitary with $\theta = 3.9\pi$. These imperfections are embedded in our simulations.

To characterize these errors we obtain the explicit form of the unitary evolution generated by the drive Hamiltonian in Eq. 3 (from the main text) and compare it with the ideal beam-splitter unitaries S_1 (Eq. 1), S_2 (Eq. 15), and the B-pulses $B(\theta)$ (Eq. 2). The Hamiltonian in Eq. 3 (from the main text) generates a unitary evolution, and the corresponding dynamics can be determined by solving

$$i\hbar\frac{\partial}{\partial t}|\psi(t)\rangle = H(t)|\psi(t)\rangle, \tag{47}$$

where $|\psi(t)\rangle$ is the state of the system at an arbitrary time t and $\Omega_{01}(t)$ ($\Omega_{12}(t)$) are the drives from Eq. 1 (main text). Following Eq. (47), we obtain the dynamics of our three-level system initialized in the computational basis states $|0\rangle$, $|1\rangle$, and $|2\rangle$. Each of these states undergo the same evolution for the same time, resulting in states $|\psi_0(t)\rangle$, $|\psi_1(t)\rangle$, and $|\psi_2(t)\rangle$ respectively. The explicit form of the corresponding unitary operator at arbitrary time t can thus be written as

$$U_{\mathrm{sim}}(t) = |\psi_0(t)\rangle\langle 0| + |\psi_1(t)\rangle\langle 1| + |\psi_2(t)\rangle\langle 2|. \tag{48}$$

This unitary operator is obtained numerically for given experimental parameters. Numerical integration is performed using the fourth-order Runge-Kutta method with a step size of (AWG sampling rate)$^{-1}$. Thus, the super-Gaussian pulse envelope of 56 ns duration is discretized by the AWG sampling rate of 1 GS/s. Deviation of U_{sim} from the ideally expected $U(t)$ ($= S_1$ or S_2 or $B(\theta)$) is calculated using the 2-norm of the difference between the operators, given by $\xi = ||U(t) - U_{\mathrm{sim}}(t)||_2$. Note that the maximum value of ξ is 2. Thus we can quantitatively assess each individual unitary operation implemented in the experiments. We have also analyzed the results of single-qutrit quantum process tomography (QPT) [5, 7] and obtained the precision of the overall pulse sequence ($S_1 B(\theta) S_1$). Process matrices (χ_{sim}) resulting from the simulation (without decoherence) including pulse errors are compared with that of the ideal process matrix (χ_{ideal}) using the fidelity measure $\mathcal{F} = [\mathrm{Tr}\sqrt{\sqrt{\chi_{\mathrm{ideal}}}\chi_{\mathrm{sim}}\sqrt{\chi_{\mathrm{ideal}}}}]^2$.

Overall, this analysis results in the following bounds for the errors. For $N = 1$ the beam-splitter unitary S_1 has $\xi = 0.01$ and the average ξ for $B(\theta)$ is 0.06. From tomography, $\mathcal{F} = 0.98$, averaged over $\theta \in [0, 4\pi]$. For $N = 2$, we have $\xi = 0.07$ for S_2 and average $\xi = 0.21$ for $B(\theta)$. Further, evaluating the whole process ($S_2 B(\theta) S_2 B(\theta) S_2$) via QPT, we obtain an average fidelity $\mathcal{F} = 0.91$. Results from the simulations with these pulse errors alongside with decoherence match the experimental datasets quite well as shown in Figs. (2,3) of the main text and Supplemetary Fig. (3).

SUPPLEMENTARY NOTE 6: REPRESENTATION ON THE MAJORANA SPHERE

Geometrical representations are useful for understanding quantum operations. Here we adopt the Majorana representation to visualize geometrically the single-qutrit dynamics during our protocol. We simulate the single-qutrit dynamics on the Majorana sphere for the case of multiple consecutive MZI setups.

In the Majorana geometrical representation, a particle with spin j is represented by $2j$ points (known as the Majorana stars) on a unit sphere (known as the Majorana sphere). Consider an arbitrary state of a spin j particle in the $|jm\rangle$ basis,

$$|\Psi\rangle = \sum_{m=-j}^{j} c_m |jm\rangle, \tag{49}$$

where c_m are the complex coefficients. The corresponding Majorana polynomial of degree $2j$ is constructed as $P_{|\Psi\rangle}(\zeta) = a_0\zeta^{2j} + a_1\zeta^{2j-1} + \cdots + a_{2j}$, with

$$a_r = (-1)^r \frac{c_{j-r}}{\sqrt{r!}\sqrt{(2j-r)!}}. \tag{50}$$

$P_{|\Psi\rangle}(\zeta) = 0$ has $2j$ roots, which can be plotted in the xOy plane. The inverse stereographic projections of each of these points with respect to the South Pole of the unit sphere give rise to Majorana stars ($\mathcal{S}_i$, $i \in [1, 2j]$). Thus, the Majorana representation of a qutrit ($j = 1$) consists of two Majorana stars. For $j = 1$ we have $m = -1, 0, 1$, and the qutrit basis $|0\rangle$, $|1\rangle$, $|2\rangle$ may be identified as $|1m\rangle \equiv |1-m\rangle$. For the state $|0\rangle$ both Majorana stars lie on the North Pole, $|2\rangle$ has both Majorana stars lying on the South Pole, while $|1\rangle$ is represented by one Majorana star on the North Pole and another one on the South Pole.

First, we consider a single Ramsey setup. We initialize a qutrit in the state $|0\rangle$ and simulate the pulse sequence for $\theta_1 = 0, \pi$. The corresponding quantum state dynamics is calculated and plotted as a dynamics of Majorana stars in Supplementary Fig. 11. Supplementary Figs. 11(a1),(a2),(a3) present the trajectory under the first beam splitter, during the evolution in the absence of a pulse, and respectively under the second beam splitter. The Majorana stars $\mathcal{S}_1(x_1, y_1, z_1)$ and $\mathcal{S}_2(x_2, y_2, z_2)$ are shown in red and blue colors, where x_i, y_i, z_i are Cartesian coordinates. To begin with, both stars lie at the North Pole, corresponding to the state $|0\rangle$. Under the effect of the first beam splitter, the Majorana star $\mathcal{S}_1$ moves in the plane $y = 0$, while $\mathcal{S}_2$ stays at the North Pole such that the qutrit attains the state: $(|0\rangle + |1\rangle)/\sqrt{2} + |2\rangle$, see Supplementary Fig. 11(a1). Further, since there is no pulse in this case, no change is observed in Supplementary Fig. 11(a2). Finally, the second beam splitter brings S_1 to the South Pole of the sphere, see Supplementary Fig. 11(a3), thus representing

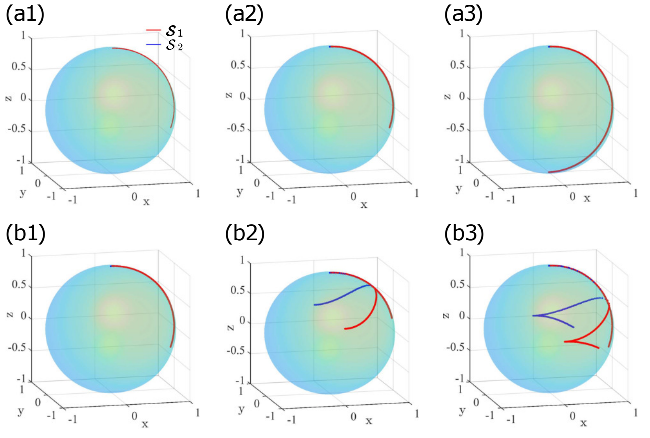

Supplementary Figure 11. Majorana trajectories at each step in the single Ramsey protocol without a pulse (a1)-(a2)-(a3) and with a pulse (b1)-(b2)-(b3) using a three-level quantum system.

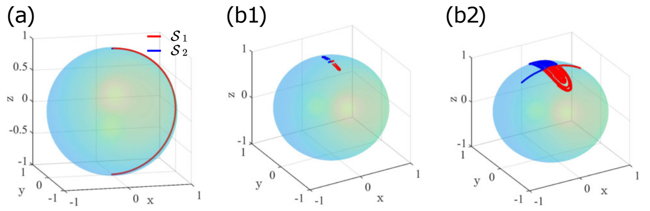

Supplementary Figure 12. Majorana trajectories resulting from the emulation of N consecutive MZI setups with Ramsey angles $\pi/(N+1)$. The subplots correspond to: (a) no pulse, (b1) $N = 100$ with 100 equal-strength pulses ($\theta_j = \pi$), and (b2) $N = 100$ with 100 pulses (θ_j =random).

the state $|1\rangle$ (one star at the north pole, one star at the south pole). The corresponding trajectories for $\theta_1 = \pi$ are shown in Supplementary Fig. 11(b1),(b2),(b3). These two cases with $\theta_1 = 0, \pi$ are clearly distinct as observed from Supplementary Figs. 11(a2) and (b2). Supplementary Fig. 11(b2) shows a non-trivial trajectory, in which $\mathcal{S}_1$ partially retraces its path very quickly and in the meantime $\mathcal{S}_2$ moves along the previous trajectory of $\mathcal{S}_1$ such that both of these Majorana stars meet somewhere in the middle of the trajectory and then start moving symmetrically in different directions. This step corresponds to the generation of the coherence between states $|0\rangle$ and $|2\rangle$. Finally, the last step implements the same beam splitter again, leading to the state $(|0\rangle + |1\rangle)/2 + |2\rangle/\sqrt{2}$ with Majorana stars $\mathcal{S}_1(0.586, 0.792, -0.172)$ and $\mathcal{S}_2(0.586, -0.792, -0.172)$. Clearly, the case $\theta \neq 0$ and $\theta = \pi$ are distinguishable by the different star constellations.

Next, we proceed with this geometrical representation and observe the single qutrit dynamics with multiple pulses. For $N = 2$, with $\theta_1 = \theta_2 = \pi$, we find that the coordinates of the final-state Majorana stars are $(0.062, \pm 0.935, 0.350)$. For $N \geq 2$, both the Majorana stars end up in the northern hemisphere. Supplementary

Figs. 12(a-b1-b2) present the final states obtained in the case of no pulse, 100 pulses with equal $\theta_j = \pi$, and 100 pulses with randomly chosen θ_js respectively. As discussed earlier, the case of no pulse corresponds to the Majorana stars $\mathcal{S}_1(0,0,-1)$ and $\mathcal{S}_2(0,0,1)$.

The final state of the single-qutrit emulating 100 consecutive Ramsey setups with 100 pulses is confined to the region around the North Pole, see Supplementary Figs. 12(b1-b2). Thus a completely contrasting configuration of the Majorana stars is observed for the case of no pulse versus the case with many pulses (here 100). In Supplementary Fig. 12(b1), where all B-pulses are of strength $\theta = \pi$, final state has both Majorana stars lying very close to the North pole. An interesting situation is seen in Supplementary Fig. 12(b2) wherein even a bad choice of arbitrary strengths of the B-pulses also correspond to a Majorana trajectory that is found to stay close to the North pole. An example showing the average Majorana trajectory with arbitrary B-pulse strengths for $N = 25$ is shown in Fig. 7 of the main text. Thus it is clear from the Majorana geometrical representation that for the case of large number of pulses, the probability of interaction-free detection is quite high and that the B-pulse strength does not matter anymore.

To conclude, we obtained the signature of coherent interaction free detection on the Majorana sphere. The results obtained from the single qutrit dynamics on the Majorana sphere is in complete agreement with the theoretical expectations and simulations.

[1] P. Kwiat, H. Weinfurter, T. Herzog, A. Zeilinger, and M. A. Kasevich, Phys. Rev. Lett. **74**, 4763 (1995).

[2] P. Kwiat, H. Weinfurter, T. Herzog, A. Zeilinger, and M. A. Kasevich, Phys. Rev. Lett. **74**, 4763 (1995).

[3] P. G. Kwiat, A. G. White, J. R. Mitchell, O. Nairz, G. Weihs, H. Weinfurter, and A. Zeilinger, Phys. Rev. Lett. **83**, 4725 (1999).

[4] R. H. Brown and R. Q. Twiss, Nature **177**, 27 (1956).

[5] M. A. Nielsen and I. L. Chuang, *Quantum Computation and Quantum Information* (Cambridge University Press, Cambridge UK, 2000).

[6] P. Neumann, J. Beck, M. Steiner, F. Rempp, H. Fedder, P. Hemmer, and F. Jelezko, Science (New York, N.Y.) **329**, 542 (2010).

[7] S. Dogra and G. S. Paraoanu, AIP Conference Proceedings **2362**, 03004 (2021).
